# Tomographic brain imaging with nucleolar detail and automatic cell counting

**DOI:** 10.1038/srep32156

**Published:** 2016-09-01

**Authors:** Simone E. Hieber, Christos Bikis, Anna Khimchenko, Gabriel Schweighauser, Jürgen Hench, Natalia Chicherova, Georg Schulz, Bert Müller

**Affiliations:** 1 Biomaterials Science Center, Department of Biomedical Engineering, University of Basel, Gewerbestrasse 14, 4123 Allschwil, Switzerland; 2 Institute of Pathology, Department of Neuropathology, University Hospital of Basel, Schönbeinstrasse 40, 4001 Basel, Switzerland; 3 Medical Image Analysis Center, Department of Biomedical Engineering, University of Basel, Gewerbestrasse 14, 4123 Allschwil, Switzerland

## Abstract

Brain tissue evaluation is essential for gaining in-depth insight into its diseases and disorders. Imaging the human brain in three dimensions has always been a challenge on the cell level. *In vivo* methods lack spatial resolution, and optical microscopy has a limited penetration depth. Herein, we show that hard X-ray phase tomography can visualise a volume of up to 43 mm^3^ of human *post mortem* or biopsy brain samples, by demonstrating the method on the cerebellum. We automatically identified 5,000 Purkinje cells with an error of less than 5% at their layer and determined the local surface density to 165 cells per mm^2^ on average. Moreover, we highlight that three-dimensional data allows for the segmentation of sub-cellular structures, including dendritic tree and Purkinje cell nucleoli, without dedicated staining. The method suggests that automatic cell feature quantification of human tissues is feasible in phase tomograms obtained with isotropic resolution in a label-free manner.

From Cajal’s publication in 1888^1^ the cerebellum has been subject to investigations seeking to correlate brain microanatomy with function^2^. Magnetic resonance imaging (MRI) is the current standard for *in vivo* studies, while functional MRI (fMRI) and diffusion tensor sub-modalities enable the extraction of functional information and the identification of neuronal paths, respectively^3^. Positron emission tomography (PET) also reveals functional information, for instance in the investigation of the cerebellum’s involvement in neuropathic pain^4^. Contrast agents are required for PET imaging and MRI^5^. While *in vivo* methods have significantly improved only recently, *ex vivo* techniques currently remain the only option for reaching sub-cellular resolution when the imaging depth moves beyond light microscopy range. The predominant method is histology that have been showing progress in microscopy, such as the optimisation of optical coherence scanning, confocal microscopy and light sheet illumination^6^,^7^,^8^. It allows for the three-dimensional visualisation of tissue slices more than 100 micrometers in thickness with or without serial sectioning^6^,^7^,^8^. In the case of the cerebellum, such approaches have been applied to provide insights into the three-dimensional cerebellar structure and the changes during its development^6^,^7^,^8^. More recently, a specimen preparation technique was developed to render opaque tissues transparently, thereby providing the three-dimensional visualisation of, for example, murine brain samples^9^. Shortcomings of the clearing methods include technical demand, tissue consumption, a restriction on antibody-based labelling, the limited storage time of the sample and the low amount of individual stainings that can be applied to a single specimen. Since the discovery of cells, visual inspection has been a common investigation method for localisation and analysis purposes. In the past few years, increasing numbers of automatic cell and nuclei segmentation methods have been developed for localization and analysis purposes, particularly in cytology^10^,^11^. However, most contemporary approaches are restricted to two dimensions.

Automated neuron segmentation approaches can be categorized into global and local ones as described in reviews^12^,^13^. In global methods fore- and background separation is followed by skeletonization. Information from the entire image is assessed towards an initial reconstruction, usually by global thresholding or edge detection algorithms. A refinement process follows, where the initial reconstruction is thinned in an iterative way. Global methods are computationally expensive, because the complete image has to be considered, even if the region of interest covers only a fraction. Recent advances include the use of curvelet pre-processing and multi-scale image treatment prior to skeletonization^14^, as well as DF (distance-field) tracing approach, to skeletonize the neurons based on two thrust-pressure force fields^15^. In local methods, the image is investigated around initial seeding points that are manually or automatically selected. Afterwards a minimal energy method such as snake or watershed segmentation is used to trace the neuronal path, *cf.* for example^16^,^17^,^18^. Local methods are challenged by branching points or crossing neurites, and the automatic selection of seeding points is not a trivial task. Tracing dendrites or axons beginning from the cell body has thus been proposed^19^ also for images with low signal-to-noise ratio^20^. A prevalent example of a combination of the global and local approach is the APP (All-Path Pruning) algorithm^21^ and recent developments thereon^22^,^23^. All mentioned methodologies are designed for microscopy data sets, where the neural structures of interest are labelled and the remaining tissue components form the background. Thus, they are not suitable for X-ray phase contrast tomograms.

Efforts in three-dimensional data acquisition most frequently involve confocal microscopy. They remain limited to a specimen thickness below 200 μm^6^. In computational pathology, automatic evaluation has not yet achieved diagnostic accuracy for most applications^24^, with cytological specimen analysers used for pre-screening of cervical smear specimens^25^. Within this context, X-ray phase contrast tomography promises to provide a solution for the three-dimensional visualisation and quantification of tissues embedded for histological examination prior to sectioning^26^,^27^. Typical specimen diameters range from 6 to 10 mm. Recently, X-ray phase tomography has provided sufficient image contrast in biomedical imaging, microscopy and materials science (ref. 28 and refs. therein). Measuring the real part of the refractive index offers an efficient technique featuring sub-micron resolution in three dimensions, without the use of staining or contrast agents, in analogy to differential interference contrast, a method employed to visualise sub-cellular features in thin layers of live cells through the use of polarised light^29^. The method has already been applied to the three-dimensional visualisation of Purkinje cells in the cerebellum^26^ and the detection of breast cancer brain micrometases, with the prospect of *in vivo* application^30^. Herein, we present the first three-dimensional visualisation and quantification of the human cerebellum with sub-cellular resolution down to the Purkinje cell nucleolus, by using phase contrast. Within the isotropically resolved volume we were able to detect and segment several thousand Purkinje cells, without labelling or using contrast agents. A sub-micron resolution measurement revealed the dendritic tree of Purkinje cells as well. The results were validated by conventional histology.

## Results

### Feature identification based on histology

**Figure 1:**
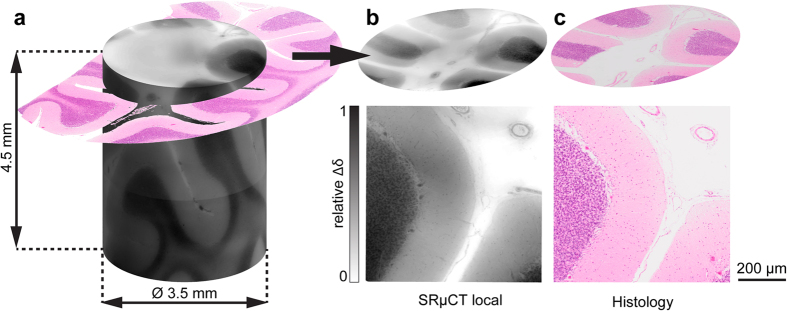
2D-3D registration and comparison of registered CT slice vs. histological slice. (**a**) Identification of the histological section within the CT data set. (**b**) CT slice and selected region mapped to (**c**) histological section. The automatic 2D-3D registration provides the identification of the *Stratum granulosum* and *Stratum moleculare* with comparable intensities, as well as the location of the Purkinje cells (see also Supplementary Video 1).

Human cerebellum specimens were measured using synchrotron radiation-based micro computed tomography (SRµCT) in local phase contrast mode, and they were subsequently histologically sectioned for validation. Figure 1 shows a direct correlation between the registered computed tomography (CT) slice (Fig. 1b) and the respective histological section (Fig. 1c) in terms of cerebellar tissue layers and cell boundaries. The three-dimensional dataset covers a cylindrical volume of 43 mm^3^ with an isotropic voxel length of 1.75 μm, and it is located inside a cylindrical formalin-fixed paraffin-embedded specimen. Figure 1a illustrates the location of the histological slice in the CT data, automatically found using 2D-3D registration. The relative intensities match in both modalities. The phase contrast tomography dataset shows minor local gradients, while the histological slice shows a variation in overall colour intensity, due to common staining inhomogeneity.

**Figure 2:**
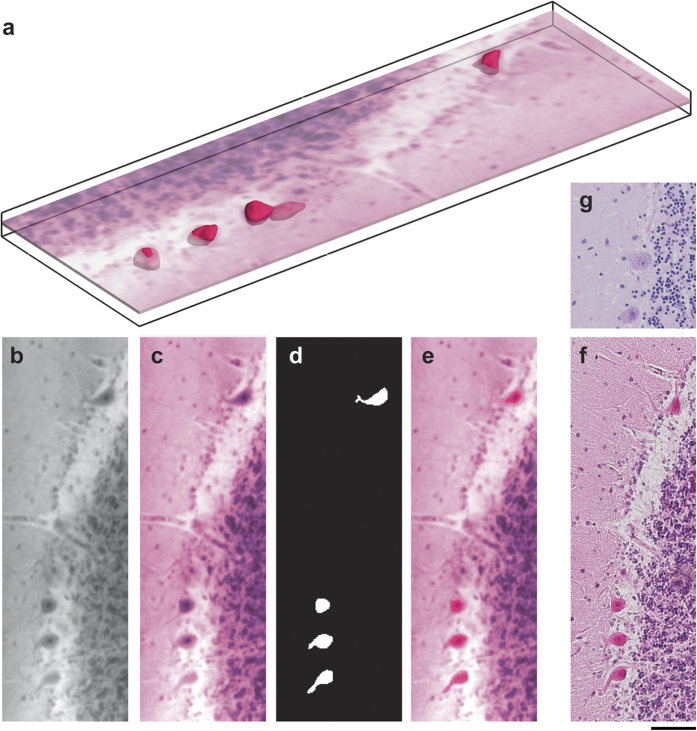
Localisation of the Purkinje cells using histological findings and its extension into the third dimension. (**a**) 3D view of Purkinje cells with a tomogram slice resembling a 3D extension of histology and (**b**–**g**) their identification. (**b**) Registered phase contrast image. (**c**) Phase contrast image coloured similar to H&E staining. (**d**) Purkinje cell segmentation mask. (**e**) Phase contrast image with separately coloured Purkinje cells to resemble f, H&E histology. The staining is insufficient to visualise the nucleolus, due to *post mortem* autolysis. g, Biospy - H&E section with nucleoli visible. The scale bar corresponds to 100 µm (see also Supplementary Video 2).

Figure 2a displays a selection of 3D Purkinje cells which were automatically identified, validated by histological findings (Fig. 2b–e) and finally extended into the third dimension. The Purkinje cells detected by applying a Frangi-based filter, and fully segmented using subsequent region growing (Fig. 2d), match well with their corresponding features found within the histological slice, using automatic registration (Fig. 2f). The original CT slice (Fig. 2b) is recoloured without and with the separate consideration of Purkinje cells (Fig. 2c,e, respectively), in order to resemble the histological slice and to emphasise the two samples’ correspondence. The slice in the three-dimensional image (Fig. 2a) represents the location and the thickness of the histological section in comparison to the volumetric Purkinje cells using the CT dataset in isotropic spatial resolution. The extension of Purkinje cells identified in the registered phase contrast slice was performed within a box measuring 0.70 mm × 0.23 mm × 0.03 mm. The cells were detected by Frangi-based filtering, and their morphology was segmented using a region-growing approach. Haematoxylin and eosin staining is not optimised for the visualisation of Purkinje cell structures. The nucleoli of the Purkinje cells are hardly visible in the histological slide, which is likely the result of *post mortem* autolysis. Nonetheless, the anatomical microstructures remain detectable.

### Quantification of the Purkinje layer

**Figure 3:**
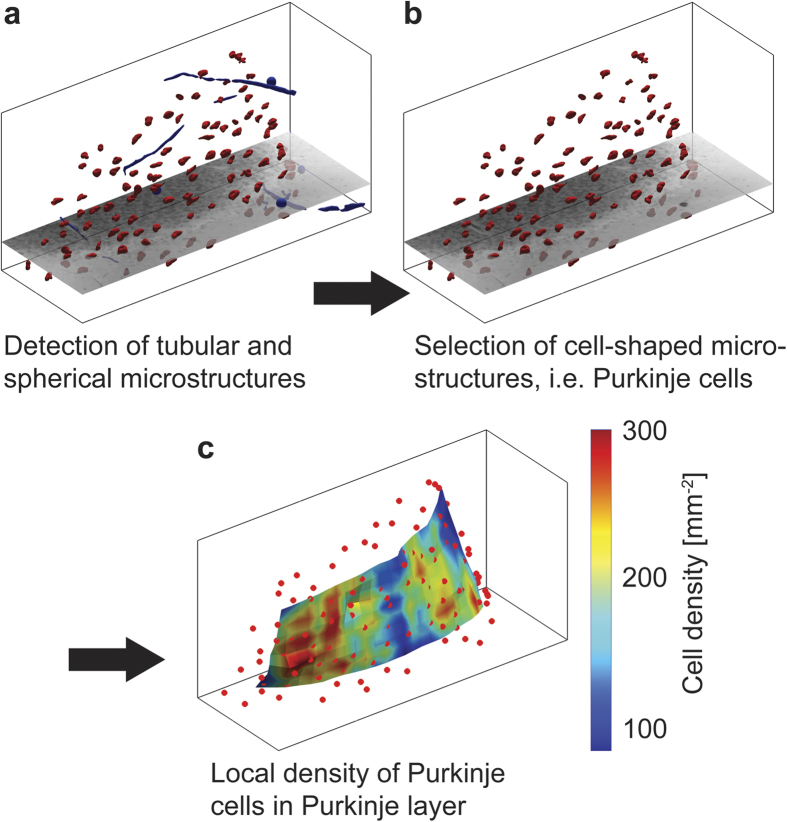
Purkinje cell layer identification in a volume of 0.4 mm × 1.0 mm × 0.5 mm. (**a**) Objects detected by the Frangi-based filter include vessels and *Corpora amylacea*. (**b**) After deselecting extremely tubular and spherical structures, the error rate was determined based on approximately 100 objects. (**c**) The Purkinje cell layer is coloured according to local cell density, shown with cell locations (red). The cell density was evaluated with respect to surface area and varied between 90 and 290 cells per mm^2^. Average cell density was 177 cells per mm with respect to the Purkinje cell layer (see also Supplementary Video 3).

In the present study the Purkinje cell layer and related quantities were determined based on cell positions, as illustrated in Fig. 3, rather than by extracting the separation layer between the *Stratum moleculare* and *Stratum granulosum*. The feature-based filter adapted to the geometry of Purkinje cells selects all tubular and spherical microstructures within the specified range, including blood vessels and *Corpora amylacea*. After selecting elliptically shaped objects, 108 objects were automatically detected as cells within a volume of 0.2 mm^3^. A comparison, using visual inspection, led to three false positives and two negative trues. Thus, the error of Purkinje cell localisation corresponds to 5%. Visual inspection was performed on 300 tomography slices and two histological slices. The histological slices were registered within a ROI_1_ sized 600 × 220 × 300 voxels and used to match the counterparts of the Purkinje cells in the tomographic data set. The criteria to identify further Purkinje cells in the tomographic ROI_1_ were the size, the morphology and the characteristic gray-value intensity of the nucleolus. In addition, we examined the detection error based on four histological slices and obtained an error of 11% considering the 61 Purkinje cells of the histological slices and their counterparts. The analysis is restricted to a region where the intensity change is less than a half compared to ROI_1_.

**Figure 4:**
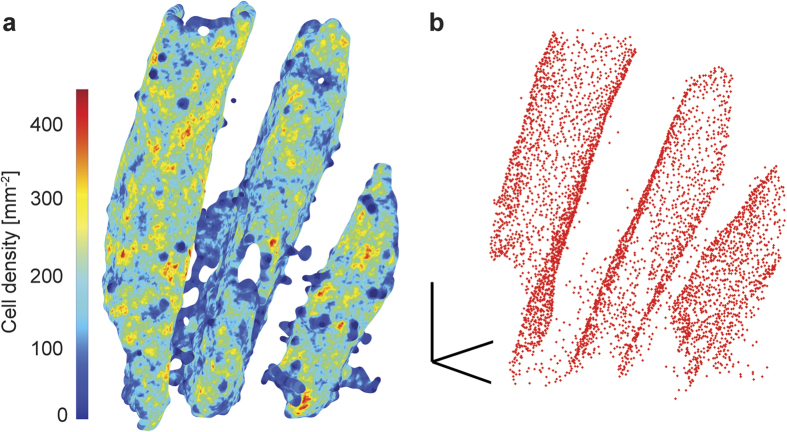
Purkinje layers coloured according to the local density of Purkinje cells and their location in a volume of 43 mm^3^. (**a**) The average detected density was 165 cells per mm^2^, related to the manifold and volumetric cell density to 116 mm^−3^ of (**b**) localised Purkinje cells. Segmented objects were filtered in sparse distributions not connected to the Purkinje layer, which is derived and described implicitly using a level set approach. It features disconnectivities at the border of the dataset. The axes of the scale bar correspond to 1 mm (see also Supplementary Video 4).

Figure 4 shows the Purkinje cells of the acquired dataset and the Purkinje layer colour-coded by local cell density. The layer forms the characteristic shape of the interface between the *Statum granulosum* and *moleculare*. The average segmented volume of the approximately 5,000 formalin-fixed paraffin-embedded Purkinje cells is 4,850 μm^3^.

The computational time for the cell identification of one height step of size 1500 × 2400 × 2400 is 28.2 hours by means of a laptop computer (Intel^®^ Core^TM^ i7-4600U CPU @ 2.10 GHz up to 3.3 GHz, 8 GB RAM). The time is reduced to 1.0 hour on a computing cluster with 48 cores (Intel^®^ Xenon X5650 @ 2.67 GHz, 8 GB RAM). The computational effort is approximately a factor of 10 less expensive than a multi-scale neuron segmentation approach presented very recently by Hernandez-Herrera, P. *et al*.^31^.

### Purkinje cell with a dendritic tree

**Figure 5:**
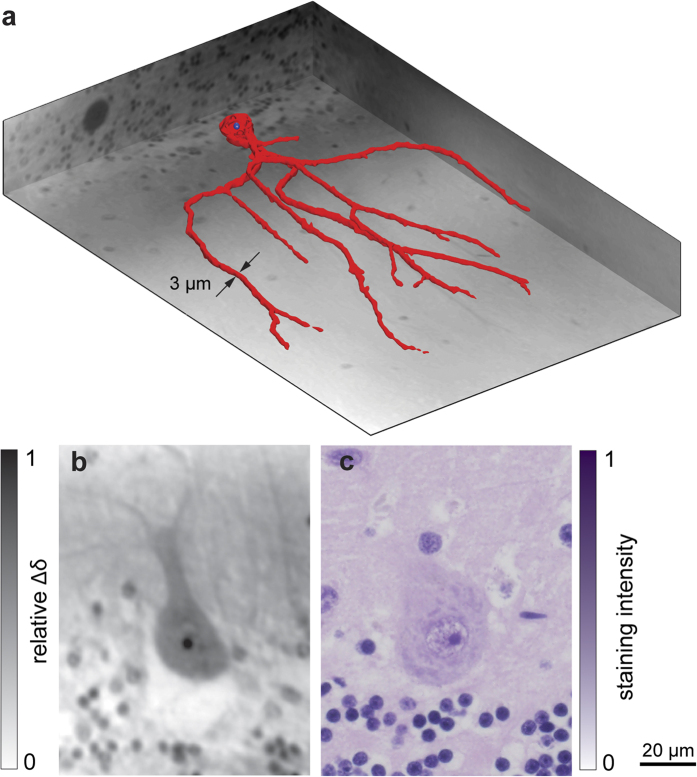
Purkinje cell including the main dendritic tree. (**a**) Representation as a 3D surface and (**b**) a 2D phase tomogram slice in comparison to (**c**) a H&E stained histological image of a Purkinje cell. The images verify clear correspondence with respect to cell morphology and the nucleolus, and it is less visible in the nucleus. In the 3D view the red-coloured surface represents the outer contour of the segmented cell and the blue one the nucleolus. The dimensions of the region of interest, namely 382.5 × 247.5 × 54.0 μm^3^, result in 56.1 million isotropic voxels with an edge length of 0.45 μm (see also Supplementary Video 5).

The dataset recorded with sub-micron resolution from the same cerebellum not only confirms our findings concerning the identification of Purkinje cells, but it also indicates the spherical boundary of the nucleus, as shown in Fig. 5b. The haematoxylin and eosin staining of the histological slice (Fig. 5c) reveals the nucleolus in a typical Purkinje cell in a manner that would be suitable for manual cell counting. The microscopic image provides sub-micrometer resolution laterally but covers approximately 4 μm in the axial direction. Thanks to the isotropic resolution of the phase contrast dataset, the dendritic tree is significantly more recognisable in the shown CT slice. The three-dimensional extension of the findings is performed using Frangi filtering and yields the segmentation of the complete cell, including a major part of its dendritic tree (Fig. 5a). Signal-to-noise values of 13 and 5 were obtained for the data sets acquired at ESRF, Grenoble, France, and Diamond Light Source, Didcot, UK, respectively, *cf.* Method section.

## Discussion

As shown above, phase tomography enables the investigation of three-dimensional microstructures in brain tissue, reaching down to sub-cellular spatial resolution. The visualisation of individual Purkinje cells fixed in formalin has already been accomplished by Schulz *et al*.^26^, using hard X-ray phase contrast tomography, based on grating interferometry with a pixel size of 5.1 μm and an estimated spatial resolution of 20 μm. As the spatial resolution of grating interferometry is currently limited to gratings’ periodicities of several micrometers, a spatial resolution of about 1 μm can only be achieved using in-line phase contrast techniques. To overcome the issue of the lower contrast of single-distance phase contrast tomography in comparison to grating interferometry^32^, brain tissue was embedded in paraffin. The reduction of the pixel size to one-third the size employed in the previous study was necessary to visualise the 3 μm-thick nucleoli.

Conventional histological embedding exchanges water and most lipids for wax, a mixture of paraffin and polymers^33^. The percentage of processing-induced shrinkage is expected to be high and can be estimated at approximately 30% according to its original size^34^. After shrinking by 30%, tissue contrast is expected to increase by approximately 40%. Furthermore, potential small-scale deformations, caused by the rotation of the specimen, are suppressed by paraffin wax solidification.

Andersen *et al*.^35^ estimated the density of Purkinje cells as being 810 cells per mm^3^, while we found a density of 116 mm^−3^ in the 43 mm^3^ specimen, covering about 1/3000 of an average human cerebellum. However, when extrapolating the relationship of Purkinje cell density during ageing, presented in a recent study^36^, a human within the considered age range would exhibit about 130 cells per mm^3^, which is in agreement with our result. It has to be noted that volumetric Purkinje cell density depends strongly on the choice of the considered volume, as large neurons form thin layers only. Thus, density with respect to the area of the cell layer leads to more reliable characterisation within small volumes. Provided a sufficient amount of beam time, a scan would be feasible for a complete cerebellum separated into parts using the present approach. Assuming a human brain with a volume of 1.3 dm^3^ and a cerebellum with approximately 10% of its volume, 6,500 scans would be necessary to cover the entire cerebellum with a pixel length of 1.75 μm, using a 2000 × 2000 detector with volumes of approximately 20 mm^3^. A scan time of five minutes–as achieved in our last experiments–leads to a necessary beam time of 23 days in total. Before the scans, the cerebellum should be embedded in paraffin and cut into rod-like blocks on a basis of about 2.5 × 2.5 mm^2^.

Cell morphology, including the nucleolus, is readily visible in both datasets with pixel sizes 1.75 and 0.45 μm (see Figs 2 and 5). Interestingly, the nucleolus is only just about recognisable in the mapped histological slice, as shown in Fig. 2, most probably due to autolysis that starts immediately after death and is thus a common challenge in autopsy histology. Particularly after death, the chromatin is affected within hours^37^.

The results show that the nucleolus reveals a significantly larger phase shift, which indicates the higher electron density of the nucleolus in comparison to its surroundings. Nucleoli are compact structures composed mainly of RNA and proteins. They are observable in monolayers under a phase-contrast microscope^38^. It is already known that the density of the nucleolus is double the remaining nucleus and the cytoplasm in *Xenopus* oocytes^39^. Similar results were obtained in human cells at a laser wavelength of 633 nm^40^.

Generally, the present methodology can provide better insights into various morphological features that have been revealed by neuroimaging for cerebellar hypoplasia, cerebellar agenesis, pontocerebellar hypoplasia, cerebellar dysplasia, cerebellar dysmorphia and cerebellar atrophy (see also^41^ and refs. therein). It is expected to master quantification tasks, including volume measurements of an animal model cerebellum, its cellular layers, or its adjacent microstructures, isotropic cell counting of different cell types in the entire tissue volume and the investigation of cellular morphology. In particular, the loss of Purkinje cells is characteristic of diseases and disease models that include essential tremors^42^, Cockayne syndrome^43^, experimental autoimmune encephalomyelitis^44^, PGC-1a knock-out mice^45^, leaner mouse models^46^, neuronal nitric oxide synthase deficient mouse models^47^, paraneoplasmic cerebellar degeneration^48^, and a three-factor autism model^36^,^49^. Within this context, we see large potential application of the proposed method in a) developmental and morphology studies, b) disease studies and disease model development and c) the evaluation of possible therapeutic interventions. Note that the complete murine cerebellum can be scanned in non-local phase contrast tomography and that mouse Purkinje cells are only approximately 30% smaller than their human counterparts.

Purkinje cells are among the largest cells in the human body. Additionally, they occur within a distinct anatomical location, i.e. the cerebellar Purkinje cell layer. On one hand these characteristics facilitate automatic counting. On the other hand the Frangi-filter is hindered by the elliptical shape. It is expected that the proposed procedure can be likewise applied to count other cell types such as astrocytes and pyramidal neurons. Automatic counting of Purkinje cells might be affected by anatomical features at a higher spatial resolution. For example, the neurons in the *Stratum granulosum* show a dense distribution of clusters which might be mis-detected as Purkinje cells. Resampling the data to the pixel or voxel size used in the present study, however, would circumvent such complicacy. Our segmentation task differs from the usual neuron segmenting problem, where from early on, the images had a relatively high signal-to-noise ratio. e.g. by means of fluorescence microscopy^50^,^51^. It could be argued that our task is more similar to the automated segmentation of MRI images for neuronal tractography with a low signal-to-noise ratio and high structure density. Hence, segmentation approaches such as the one presented in ref. 52 could be considered, although they need the incorporation of complex anatomical knowledge, and the extrapolation of findings is problematic given the entirely different problem size scales. We exploited global and local methods’ complementarity, using Frangi filtering on the entire dataset to find seeding points for subsequent region-growing, comparable in part to the Tree2Tree algorithm^53^.

Our approach proposes how to automatically identify nucleoli in brain tissue volumes of more than 40 mm^3^. As described in recent literature, analogous tasks are often performed by manually counting within the field of view. In detail, the frequency of binucleate cells in human cerebellar tissue from patients with multiple sclerosis was examined *post mortem* and found to be increased with respect to controls^54^. The methodology presented features two major challenges. Firstly, the frequency of binucleate cells was given with respect to total Purkinje cell numbers, albeit not assessing the volume density parameter of either population. Secondly, a total of 20,000 cells needed to be counted manually, and then the prime binucleate candidates were imaged in 3D, in a copious and time-consuming two-step procedure. Using the proposed approach, the cellular and sub-cellular features in question could be investigated in the specimen automatically and efficiently, in one 3D imaging step and without any sectioning or staining. Such an approach could also benefit other analogous tasks, such as, for example, the investigation of nucleolar size as a possible prognostic characteristic for specific types of malignancies^38^,^55^. Since the nucleolus comprises packed proteins besides RNA molecules^38^, it is expected that phase contrast imaging will allow for the identification of protein aggregates caused by neuronal disorders such as Alzheimer’s disease.

Phase contrast micro-tomography not only assures backwards compatibility, but also synergies with other methods and modalities can be achieved, offering the opportunity for multimodal data analysis. The three-dimensional information obtained for the entire sample can be exploited to investigate brain tissue at sub-cellular resolution in health and disease. Scanning times only account for a fraction of the time that would have been needed for imaging based on serial sectioning, while they also avoid mechanical deformations and tissue loss due to preparation. Furthermore, inherent in destructive methods, sampling errors are also taken out of the equation. In comparison to microscopy methods after optical clearing, our approach is perfectly compatible with subsequent traditional histological sectioning and staining, including immunochemistry methods. It is also a method that preserves the chemical composition and physical properties of the examined sample to the greatest extent possible, given that the scanning of fresh samples in phosphate-buffered saline (PBS) has also been proven to provide satisfactory results^27^. Last but not least, copious tissue preparation is not necessary, given that fresh, formalin-fixed or paraffin-embedded samples can be used, without inconsistencies and limitations due to labelling techniques^56^. The procedure can be applied to further tissue types, such as the hippocampus, and larger pieces by stitching together local tomography datasets.

In conclusion, phase contrast tomography reveals sub-cellular structures of paraffin-embedded brain tissue, enables their three-dimensional quantification for scientific purposes and possibly complements medical diagnostics. Since paraffin embedding has served as a standard approach for tissue conservation for more than a century, considerable archives of formalin-fixed paraffin-embedded specimens can be examined using the proposed method, being also applicable to other soft tissues. The proposed methodology enables the identification of Purkinje cells based on automatic filtering. Thus, the study shows that automatic cell feature quantification of human tissues is feasible based on synchrotron radiation-based micro computed tomography. For the identification of structures over various length scales it could be beneficial to follow approaches such as the multi-scale neuron segmentation based on morphological filtering^31^.

## Methods

### Specimen preparation and histology

*Post mortem* specimens of a human cerebellum were excised from the donated brain of a 73-year-old male. Informed consent for scientific use was obtained. All procedures were conducted in accordance with the Declaration of Helsinki and approved by the Ethikkommission Nordwestschweiz. Specimens were fixed in 4% histological-grade buffered formalin, dehydrated in ethanol, transferred to xylenes and embedded in a paraffin/plastic polymer mixture (Surgipath Paraplast, Leica Biosystems, Switzerland). Two cylindrical fragments with a height of 4 mm were excised from paraffin blocks measuring 2.6 and 6.0 mm in diameter. After data acquisition the tissue was re-embedded for histological sectioning with a thickness of approximately 4 μm. The haematoxylin- and eosin-stained slides were digitised using a histological slide scanner (Olympus VS120 Virtual Slide Microscope, Japan).

### Data acquisition and reconstruction

Phase-contrast, single-distance X-ray tomography experiments were performed at two synchrotron facilities in local tomography configuration. The specimen with a diameter of 6 mm was scanned at the beamline ID 19 (ESRF, Grenoble, France)^57^ with an effective pixel size of 1.75 μm and a mean photon energy 19.45 keV. The detection system was a FReLoN 2 K CCD with 2048 × 2048 pixels. The scan with two height steps covered a cylindrical volume with a diameter of 3.5 mm and a height of 4.5 mm. Projections were acquired at a propagation distance of 80 cm with a pink beam. Images were taken at 2004 angular steps over 360 degrees with an exposure time of 1 s per raw image.

To reproduce the findings, the other specimen with a volume of 3 mm^3^ was scanned at Diamond Manchester Imaging Branchline I13-2 (Diamond Light Source, Didcot, UK)^58^. The effective pixel size was 0.45 μm and the mean photon energy 19 keV, using a pco.4000 camera (PCO AG, Kelheim, Germany) with 4008 × 2672 pixels. The reconstructed volume had a diameter of 1.6 mm and a height of 1.6 mm. At a distance of 5 cm, images were taken with a monochromatic beam and an exposure time of 8 s per raw image for 2400 equi-angular steps over 180 degrees. The phase shift profiles of the specimens were determined by single-distance phase retrieval. Phase contrast was recovered from a single projection taken at a defined specimen-detector distance, generated by the free-space propagation of X-rays^32^. Measurement sample-detector distances were selected based on the contour plot of the critical propagation distances for various pixel sizes and photon energies, as described by Weitkamp *et al*.^59^. Since the sample diameter was bigger than the detector field-of-view (FOV), measurements were performed in local tomography configurations for both beamlines. Flat-field and dark-field correction of local tomography data was performed using the software tool ANKAphase^59^ with an input parameter *δ*/*β*, i.e. the ratio of the refractive index decrement over the absorption coefficient of paraffin at the given energy and distance. Phase recovery was performed based on a single-distance, non-iterative, phase-retrieval algorithm with flat-field correlation and an input ratio. The tomographic reconstruction of the data used the filtered back-projection (FBP) method with a standard the Ram-Lak filter. Both steps were employed by in-house implementations in Matlab R2014b (Simulink, The MathWorks, Inc., USA). Radial gradients and ring artefacts were reduced prior to tomographic reconstruction, using zero-padding and a combined wavelet-Fourier filtering technique^60^, and then applied to the sinograms in the reconstruction procedure.

### Image analysis

#### Mapping to histology

To register a histological slice within the three-dimensional phase tomogram, we applied an automatic 2D-3D registration algorithm, an extension of the previous work of Chicherova *et al*.^61^,^62^. A first match was identified according to the published algorithm in a dataset covering the entire diameter of the specimen. We applied the scale- and rotation-invariant feature detector and descriptor SURF^63^ to both the tomogram and the histological slice to identify matching feature points in the 3D dataset. Plane fitting to the filtered point cloud led to an estimation of the slice position and orientation. To improve the registration, we extended the algorithm by applying dense feature detector self-similarity^64^. In contrast to SURF, the self-similarity detector calculates a descriptor vector on a regular grid of points which allowed us to take into account homogeneous areas of the tissue and embedding. After registering the found virtual CT slice with its histological counterpart in 2D, positioning of the plane was refined by reapplying the described approach with self-similarity as a feature point detector. Finally, the resulting set of plane parameters was transferred to extract the matching slice from the local tomography data set.

#### Data selection

The analysis of the individual Purkinje cells was performed on both tomograms. A region of interest was selected in each tomography dataset. One region of interest (ROI_1_) comprising a size of 600 × 220 × 300 voxels with voxel length of 1.75 μm, and a smaller one (ROI_2_) comprising a size of 850 × 550 × 100 voxels with voxel length 0.45 μm, was chosen to illustrate a representative part of the Purkinje cell layer. Tomography data were segmented using an implementation of the Frangi filter in Matlab R2014b (Simulink, The MathWorks, Inc., USA) by Kroon^65^,^66^.

#### Purkinje cell segmentation

The intensity values of the in-line tomograms overlap, so that thresholding is insufficient for segmentation. Hence, an implementation of a feature-based segmentation algorithm based on the ‘Frangi filter’ was used to identify cells and dendrites. Originally designed for the detection of vessels, the Frangi filter provides the probability that voxel belongs to a tubular or spherical structure^67^ by analysing the eigenvalues 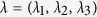 of the 3D Hessian matrix. The Frangi filter function of the scale *s* is described by:


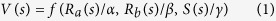


The ratio 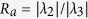 distinguishes between sheet- and tube-like structures, whilst the ratio 
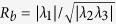
 accounts for spherical structures and for the second-order structuredness 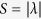. For an ideal tubular structure, the eigenvalues fulfil the conditions 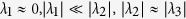
67. The constants *α*, *β* and *γ* determine the sensitivity of the filter measures *R*_*a*_, *R*_*b*_, and *S*. Finally, the measure is the maximum value of the vesselness function within a scale range given in voxels. To segment the Purkinje cells a scale range was chosen from 3 to 6 voxels (5.25 to 10.50 μm), the sensitivity parameters were *α* = 0.2, *β* = 0.5 and *γ* = 40 and objects smaller than 300 voxels (1600 μm^3^) and larger than 3000 voxels were neglected. For the dendritic tree we considered scales from 1 to 3 voxels (0.45 to 1.35 μm), sensitivity parameters *α* = 0.5, *β* = 0.1 and *γ* = 10 and chose the largest object within the region of interest. All parameters and thresholds were chosen by visual inspection, to avoid the segmentation of noise. A level set representation was used to visualise the segmented objects and to categorise their geometry^68^. *Corpora amylacea* could be identified by an absolute mean level set value larger than 12, due to their spherical shape. The ratio between the object size and the mean level set value squared was above 450 for tubular structures, thereby indicating blood vessels. Both object types were detected by the Frangi filter and sorted by the criteria detailed above. The filter was sufficiently stable despite small local gradients in the dataset. Objects on the specimen border, within a margin of 10 voxels, were neglected. To improve the outer contours of the cell the segmented area was extended using region growing, with a maximum intensity distance of 0.0385 for the segmentation mask shown in Fig. 2. Colour maps, utilised to compare virtual CT slices and histological sections, were determined by visual inspection and applied to the segmented Purkinje cells and the surrounding tissue separately after background gradient subtraction. Application of the procedure to the entire specimen resulted in spread mis-segmentations away from the Purkinje cell layer. To separate them, the segmented objects were sorted into a grid with a spacing of 50 voxels, and the largest connected set of filled cubes was selected as the domain of the Purkinje cell layer. Nucleolus location was determined by the maximal phase shift within a segmented Purkinje cell, and the nucleolus itself was segmented using gray-value thresholding according to Otsu’s method^69^ in Fig. 5.

#### Purkinje cell layer and local density

To estimate the Purkinje layer from the cell locations, we interpolated the scattered data for Fig. 3 and developed an approach based on level sets^68^ for point clouds that fold in all dimensions. The level set method describes a manifold implicitly based on a signed distance function. Note that the derivation of a smooth curve from a irregular set of points remains an active field of research for a variety of recent publications also including level sets^70^. Here, distances to the Purkinje cells were evaluated on a coarse grid with a spacing of 10 voxel lengths. After smoothing the distance transform, the zero level set was defined at a distance of 44 voxels in length. Local Purkinje cell density was computed on all grid points and projected onto the extracted manifold in a neighbourhood with a radius of 100 μm.

#### Signal-to-noise ratio

For the comparison of the signal-to-noise ratios of the data sets obtained from the two beamlines the higher resolved data set was binned four times. The binning resulted in a voxel size of 1.80 μm, comparable to the voxel size of 1.75 μm in the data set from the ESRF data. The signal-to-noise ratio SNR was derived from the *Stratum moleculare* assuming that the paraffin forms the background and using volumes comprising 600 voxels. The signal-to-noise ratio is defined by 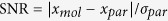, where *x*_*mol*_ represents the mean intensity value of the selected *Stratum moleculare* region, *x*_*par*_ the mean value of paraffin and *σ*_*par*_ the standard deviation of the paraffin values.

## Additional Information

**How to cite this article**: Hieber, S. E. *et al*. Tomographic brain imaging with nucleolar detail and automatic cell counting. *Sci. Rep.* **6**, 32156; doi: 10.1038/srep32156 (2016).

## Supplementary Material

Supplementary Video 1

Supplementary Video 2

Supplementary Video 3

Supplementary Video 4

Supplementary Video 5
